# Unique, Intersecting, and Overlapping Roles of C/EBP β and CREB in Cells of the Innate Immune System

**DOI:** 10.1038/s41598-018-35184-y

**Published:** 2018-11-16

**Authors:** Jason L. Larabee, Garrett Hauck, Jimmy D. Ballard

**Affiliations:** Department of Microbiology and Immunology, The University of Oklahoma Health Sciences Center, Oklahoma City, OK 73190 USA

## Abstract

CREB and C/EBP β signaling pathways are modulated during inflammation and also targeted by *Bacillus anthracis* edema toxin (ET), but how these factors individually and jointly contribute to changes in immune cell function is poorly understood. Using CRISPR/Cas9 gene editing, macrophage cell lines lacking CREB and isoforms of C/EBP β were generated and analyzed for changes in responses to LPS, ET, and IL-4. Macrophages lacking C/EBP β suppressed induction of IL-10 and Arg1, while IL-6 was increased in these cells following exposure to LPS. Examination of C/EBP β isoforms indicated the 38 kDa isoform was necessary for the expression of IL-10 and Arg1. ChIP-Seq analysis of CREB and C/EBP β binding to targets on the chromosome of human PBMC identified several regions where both factors overlapped in their binding, suggesting similar gene targeting or cooperative effects. Based on the ChIP-Seq data, a panel of previously unknown targets of CREB and C/EBP β was identified and includes genes such as *VNN2, GINS4*, *CTNNBL1*, and *SULF2*. Isoforms of a transcriptional corepressor, transducin-like enhancer of Split (TLE), were also found to have CREB and C/EBP β binding their promoter and were up regulated by ET. Finally, we explore a possible layer of C/EBP β regulation by a protein complex consisting of adenomatous polyposis coli (APC) and PKA. Collectively, these data provide new insights into the role of CREB and C/EBP β as immunosignaling regulators and targets of an important bacterial virulence factor.

## Introduction

Edema toxin (ET), the binary combination of *Bacillus anthracis* protective antigen (PA) and edema factor (EF), intoxicates cells and generates high levels of intracellular cAMP through the calmodulin-dependent adenylate cyclase activity of EF^1,2^. Studies have shown that a variety of intracellular signaling molecules (APC, Notch, GSK-3, PKA) and transcriptional regulators (TLE, CREB, C/EBPβ, RBP-J, β-catenin) are modulated in cells intoxicated by ET, yet little is known about how the collective network of ET-related targets leads to changes in immune cell function^3–6^. Based on the current understanding of these signaling molecules, their modulation could lead to a combination of both pro-inflammatory and anti-inflammatory events in ET-intoxicated cells^7–10^. Moreover, the extent to which these molecules function independently, in parallel, or intersect to drive transcriptional changes in response to cAMP remains to be defined.

ET activates signaling cascades primarily through the actions of PKA wherein cAMP binds the regulatory subunits of the PKA holoenzyme leading to the dissociation of the catalytic subunits from regulatory subunits^11^. The released catalytic subunits then phosphorylates CREB, the prototypical cAMP responsive transcription factor^12^, as well as a multitude of other cellular factors^13^. PKA activation results in transcriptional changes through both the canonical PKA/CREB axis as well as through non-canonical signaling involving PKA interfacing with factors such as those commonly associated with Wnt or Notch signaling^3–6^. As an example of non-canonical signaling, ET activates GSK-3β in the nucleus of macrophages leading to the phosphorylation of transcriptional regulators such as β-catenin and C/EBP β^3,6^. Interestingly, ET-activated GSK-3β phosphorylates CREB at Ser 129^4,14^ in addition to PKA phosphorylation at Ser 133, thus demonstrating a point of convergence between canonical and non-canonical signaling. Further study of ET-activated GSK-3β found GSK-3β phosphorylates C/EBP β within a scaffolding complex supported by adenomatous polyposis coli (APC)^6^, a large multi-domain protein important for tumor suppression and Wnt signaling. Intriguingly, increased CREB activity promotes the expression of C/EBP β^15^, which is yet another point where canonical and non-canonical signaling converges.

Despite the recent progress in dissecting the immunomodulatory activity of ET, several unanswered questions remain. For example, whether ET exploits physiologically relevant signaling events to promote immunosuppression or if ET causes aberrant signaling events leading to cellular dysfunction is not known. This stems in part from an incomplete understanding of the roles CREB and C/EBP β play in modulating immune responses. To this end, in the current study we took a multi-pronged approach to assess the general characteristics of activation of these pathways in response to both inflammatory stimuli and ET. CRISPR/Cas9 gene editing generated stable macrophage cell lines lacking CREB and isoforms of C/EBP β, and these cells were tested for changes in responses to a variety of factors. In the second part of this study, ChIP-seq analyses were performed on peripheral blood mononuclear cells (PBMC) to determine the profiles of CREB and C/EBP β localization throughout the genome. Finally, using a combination of co-immunoprecipitation approaches, we show that PKA binds and interacts with the APC complex.

## Results

### Contributions of CREB and C/EBP β to the expression of immune modulating factors

**Figure 1:**
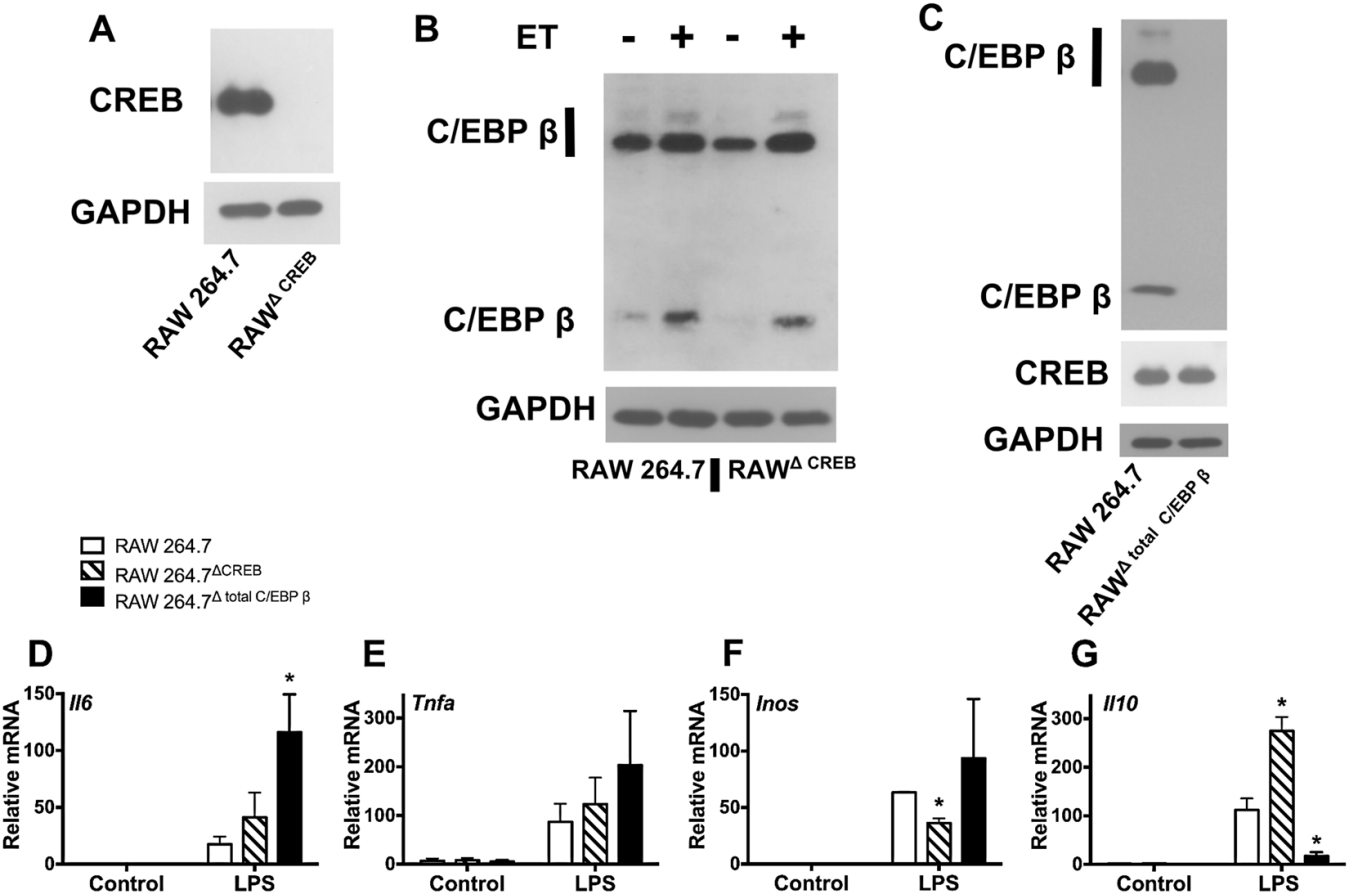
Lack of CREB or C/EBP β expression alters inflammatory responses in macrophages. RAW 264.7 cells were transduced with lentivirus containing Cas9 and gRNA directed against CREB or C/EBP β. Following transduction, clones were selected that do not express CREB (RAW^ΔCREB^) or C/EBP β (RAW^Δtotal C/EBPβ^). Panel (A) Demonstration of loss of CREB expression as shown by immunoblotting for CREB and GAPDH. Panel (B) Immunoblot analysis of C/EBP β and GAPDH expression in RAW^ΔCREB^ cells and control cells after exposure to ET (10 nM of EF and 10 nM of PA) for 6 h. Panel (C) RAW^Δtotal C/EBPβ^ cells lack C/EBP β expression as shown by immunoblot analysis of CREB, C/EBP β, and GAPDH. Panel (D–G) RAW^ΔCREB^ cells, RAW^Δtotal C/EBPβ^ cells, and control RAW 264.7 cells were exposed to 1 μg/ml LPS for 6 h. RT-qPCR was used to quantify transcript levels of *Il6*, *TNFa*, *Inos*, and *Il10*. RT-qPCR data are presented as mean (n = 3) ± S.D. Asterisks indicate significant different than control cells. *p < 0.05.

In the first part of this study, macrophage cell lines lacking CREB or C/EBP β expression were generated using CRISPR/Cas9 gene editing in RAW 264.7 cells. As shown in Fig. 1A, CREB was undetectable in cells having undergone CRISPR/Cas9 gene editing of the CREB encoding gene. Likewise, all 3 isoforms of C/EBP β were undetectable in macrophages subjected to CRISPR/Cas9-mediated editing of the C/EBP β-encoding gene (Fig. 1C). This CRISPR/Cas9 genetic manipulation yielded two stable cell lines termed RAW^ΔCREB^ and RAW^Δtotal C/EBPβ^. RAW^ΔCREB^ cells were examined for changes in levels of C/EBP β, and RAW^Δtotal C/EBPβ^ cells were examined for changes in levels of CREB. Neither transcription factor appeared to impact the expression of the other in these cells (Fig. 1B,C).

To examine the contributions of CREB and C/EBP β responses to inflammatory stimuli in macrophages, we treated RAW^ΔCREB^, RAW^Δtotal C/EBPβ^, and the parental cell line with 1 μg/ml of LPS for 6 h and then measured transcript levels from *Il6*, *Tnfa*, *Inos*, and *Il10*. As shown in Fig. 1D, relative to the parental cells *Il6* transcripts were modestly (2-fold) increased in RAW^ΔCREB^, whereas RAW^Δtotal C/EBPβ^ showed about a 7-fold increase in *Il6* transcripts. As shown in Supplementary Fig. S1, ELISA data demonstrated RAW^Δtotal C/EBPβ^ produced about 10 fold more IL-6 than parental cells after 2 to 6 h of LPS treatment. Moreover, *Tnfa* transcripts were slightly (2-fold) increased in RAW^Δtotal C/EBPβ^ and were not significantly changed in RAW^ΔCREB^ (Fig. 1E). *Inos* transcript levels were reduced in RAW^ΔCREB^, but slightly increased in RAW^Δtotal C/EBPβ^ (Fig. 1F). Finally, as shown in Fig. 1G, LPS stimulated an increase in *Il10* transcripts in RAW^ΔCREB^ macrophages whereas *Il10* transcripts were almost completely suppressed in RAW^Δ total C/EBPβ^ when compared to the parental cell line. These results suggest that C/EBP β may be more prominently involved than CREB in producing anti-inflammatory cytokines and attenuating the production of pro-inflammatory cytokines.

### C/EBP β isoforms differentially influence expression of IL-10 and IL-6

**Figure 2:**
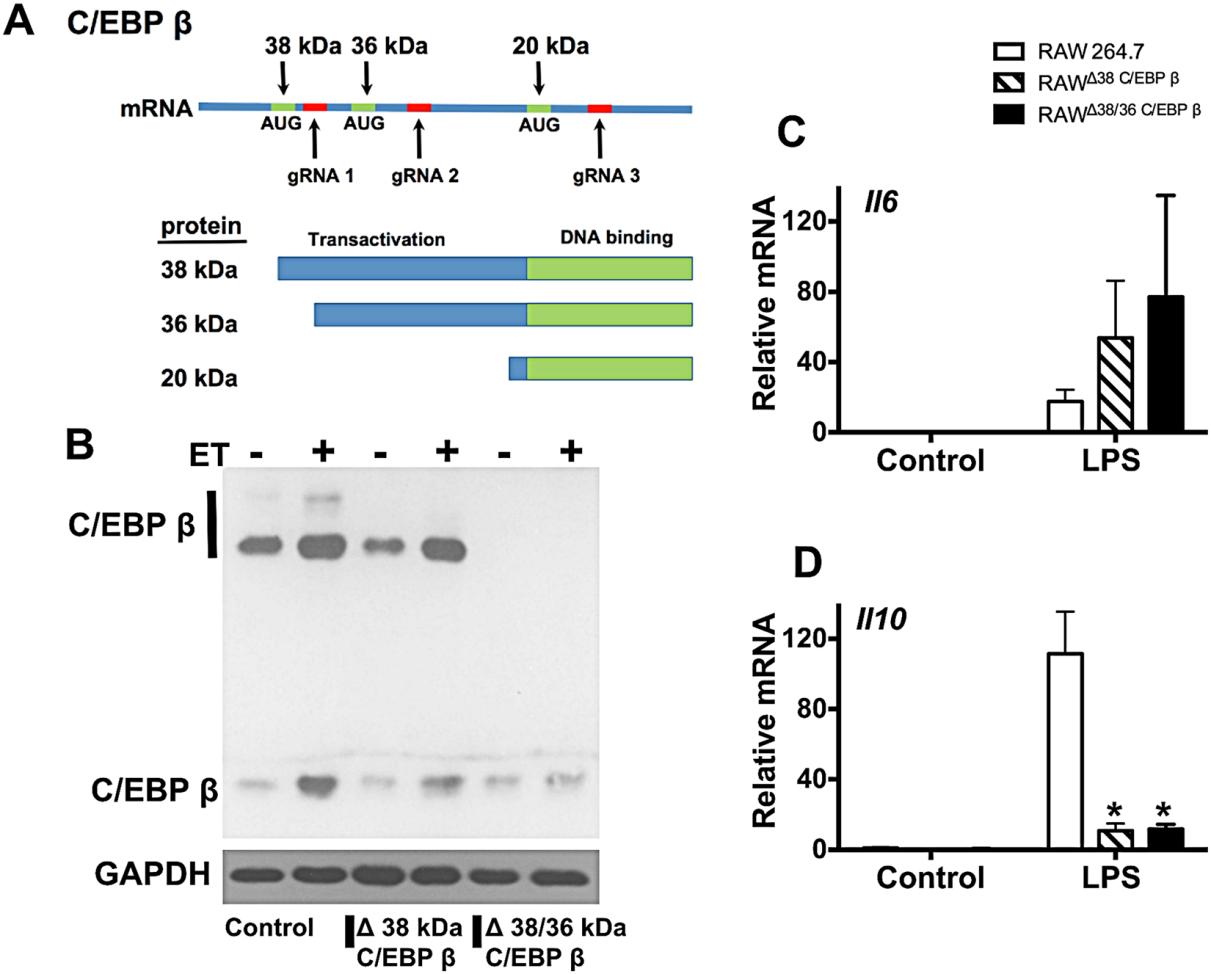
Isoform specific gene editing of C/EBP β modifies inflammatory responses in macrophages. Panel (A) Layout of the transcript that produces 3 separate protein isoforms termed 38 kDa C/EBP β, 36 kDa C/EBP β, and 20 kDa C/EBP β. The location of the 3 translation start sites (green) is depicted in relation to where the gRNAs target DNA for gene editing (red). Guide RNA 1 is used to produce a cell line lacking 38 kDa C/EBP β (RAW^Δ38 C/EBP β^). Guide RNA 2 is used to make a cell line lacking 38 and 36 kDa C/EBP β (RAW^Δ38/36 C/EBP β^). Guide RNA 3 produces a cell line lacking all 3 isoforms (RAW^Δtotal C/EBPβ^). Also presented is the domain layout of 3 C/EBP β protein isoforms showing differences in transactivation domain. Panel B–D. RAW 264.7 cells were transduced with lentivirus containing Cas9 and gRNA, and then clones were selected that do not produce 38 kDa C/EBP β (RAW^Δ38 C/EBP β^) or 38/36 kDa C/EBP β (RAW^Δ38/36 C/EBP β^). Panel (B) Demonstration of changes to C/EBP β isoform expression as shown by immunoblot analysis of C/EBP β and GAPDH. To increase expression of C/EBP β, these cell lines were exposed to ET (10 nM of EF and 10 nM of PA) for 6 h. Panel (C,D) RAW^Δ38 C/EBP β^ cells, RAW^Δ38/36 C/EBP β^ cells, and control RAW 264.7 cells were exposed to 1 μg/ml LPS for 6 h. RT-qPCR was used to quantify transcript levels of *Il6* and *Il10*. RT-qPCR data are presented as mean (n = 3) ± S.D. Asterisks indicate significant different than control cells. *p < 0.05.

C/EBP β can be expressed as 3 protein isoforms generated from a single mRNA containing 3 translation start sites (Fig. 2A). Whether each isoform has unique contributions to transcriptional responses following exposure of macrophages to LPS is not known. Each of the three isoforms share a common carboxy-terminal DNA binding and dimerization domain, but depending on where translation is initiated differ in the extent of transactivating and regulatory domains that determine binding partners and regulation (Fig. 2A). To examine these different isoforms, two additional gRNA directed towards the C/EBP β gene were chosen and used to perform CRISPR/Cas9 editing. One gRNA targeted a sequence near the first start site on the C/EBP β mRNA in order to edit out the 38 kDA form of C/EBP β. The other gRNA disrupted the second start site and produced a clone lacking both the 38 kDa and 36 kDa isoforms of C/EBP β. Isoform-specific disruption of C/EBP β was confirmed by immunoblot (Fig. 2B) and yielded two cell lines termed RAW^Δ38 C/EBP β^ and RAW^Δ38/36 C/EBP β^. As shown in Fig. 2D, RAW^Δ38 C/EBP β^ and RAW^Δ38/36 C/EBP β^ did not produce detectable *Il10* transcripts in response to LPS. RAW^Δ38 C/EBP β^ and RAW^Δ38/36 C/EBP β^ expressed elevated levels of *Il6* transcripts following LPS treatment; however, these increases in *Il6* transcripts were not as high as RAW^Δtotal C/EBPβ^ and were not statistically significant (Fig. 2C). These results indicate the 38 kDA form of C/EBP β is necessary for the induction of IL-10 while all 3 isoforms contribute to increases in IL-6.

### C/EBP β and CREB contribute to cAMP-induced expression of IL-10 and Arg1

**Figure 3:**
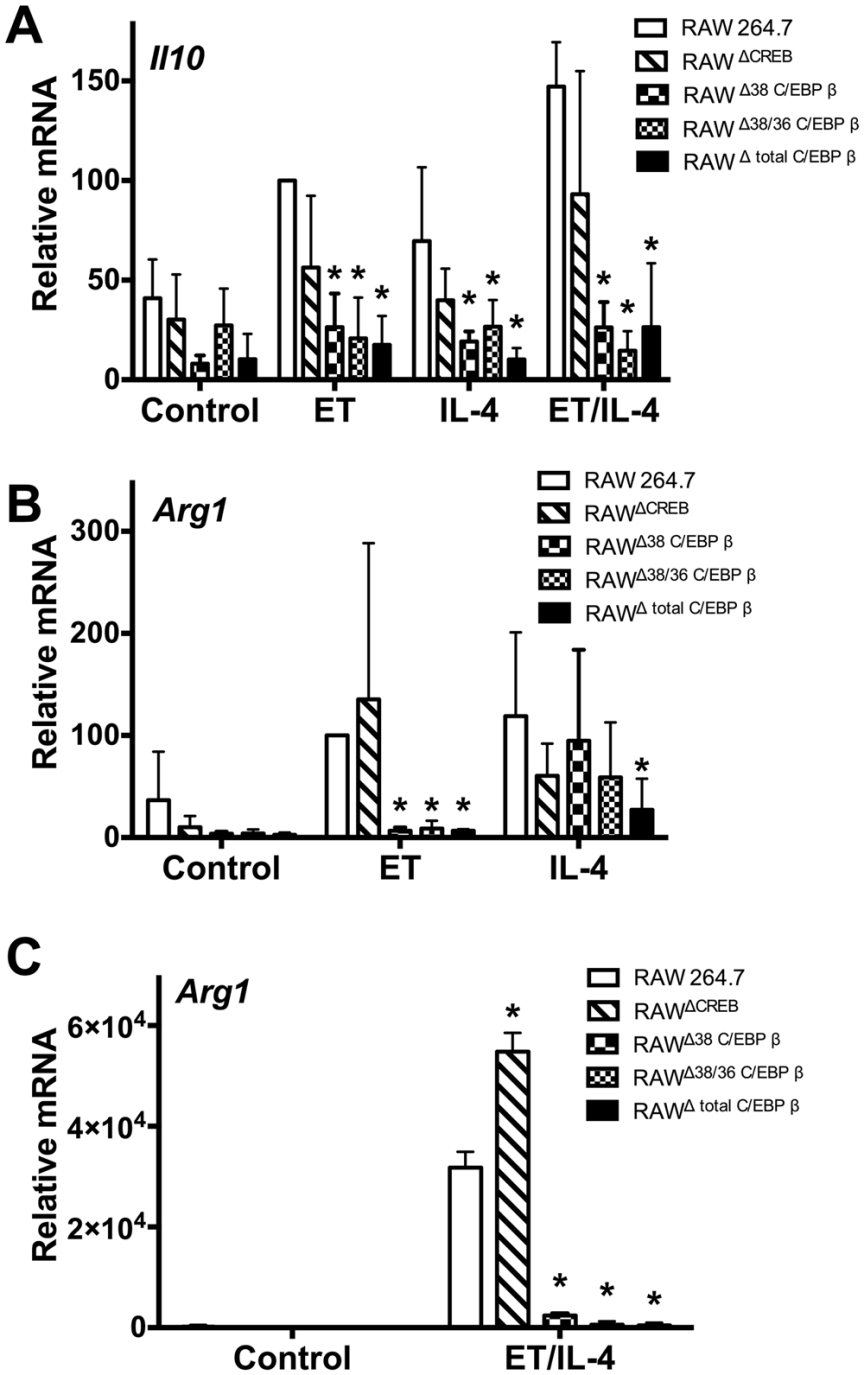
Isoforms of C/EBP β effect cAMP responses in macrophages. Panel (A–C) RAW^ΔCREB^ cells, RAW^Δtotal C/EBPβ^ cells, RAW^Δ38 C/EBP β^ cells, RAW^Δ38/36 C/EBP β^ cells, and control RAW 264.7 cells were exposed for 24 h to ET (10 nM of EF and 10 nM of PA), IL-4 (20 ng/ml), or a combination of these factors. RT-qPCR was used to measure transcript levels of *Il10* and *Arg1*. These data are presented as mean (n = 3) ± S.D. Asterisks indicate significant different than control cells. *p < 0.05.

Next, experiments examined how C/EBP β isoforms contributes to anti-inflammatory responses induced by stimuli such as IL-4 and ET. IL-10 production was analyzed in the CRISPR/Cas9 edited RAW 264.7 cells exposed to ET, IL-4, or a combination of ET and IL-4. As shown in Fig. 3A, *Il10* transcript levels in RAW^ΔCREB^ were reduced by about 25% compared to the parental RAW 264.7 cells. Evaluation of RAW^Δtotal C/EBPβ^ and the isoform specific knockout cell lines (RAW^Δ38/36 C/EBP β^ and RAW^Δ38 C/EBP β^) revealed that *Il10* transcript levels were suppressed in each treatment condition by approximately 75% (Fig. 3A).

Arg1 is a marker of anti-inflammatory M2 macrophages and we were curious to know if CREB and/or C/EBP β might influence the expression of Arg1. As shown in Fig. 3B, treatment with ET increased levels of *Arg1* transcripts in RAW 264.7 and this was further enhanced in RAW^ΔCREB^ cells. Conversely, *Arg1* transcript levels were nearly undetectable in RAW^Δ38 C/EBP β^, RAW^Δ38/36 C/EBP β^, and RAW^Δ total C/EBP β^ cells treated with ET (Fig. 3B). To determine if this effect was unique to treatment with ET, *Arg1* transcript levels were also measured in cells treated with IL-4. Arg1 levels were not repressed in RAW^ΔCREB^, RAW^Δ38 C/EBP β^ and RAW^Δ38/36 C/EBP β^, but were reduced by almost 80% in RAW^Δ total C/EBP β^ cells (Fig. 3B). In agreement with work from other groups^16^, the combination of IL-4 and ET resulted in a synergistic increase in Arg1 (Figs. 3B,C). Following the trend of the ET only treatment, Arg1 induced by ET/IL-4 was abolished in all C/EBP β knockout cell lines (RAW^Δ38 C/EBP β^, RAW^Δ38/36 C/EBP β^, and RAW^Δ total C/EBP β^), while a slight increase in Arg1 was observed in RAW^ΔCREB^ (Fig. 3C).

### C/EBP β binds IL-6 and IL-10 genes in human PBMC

**Figure 4:**
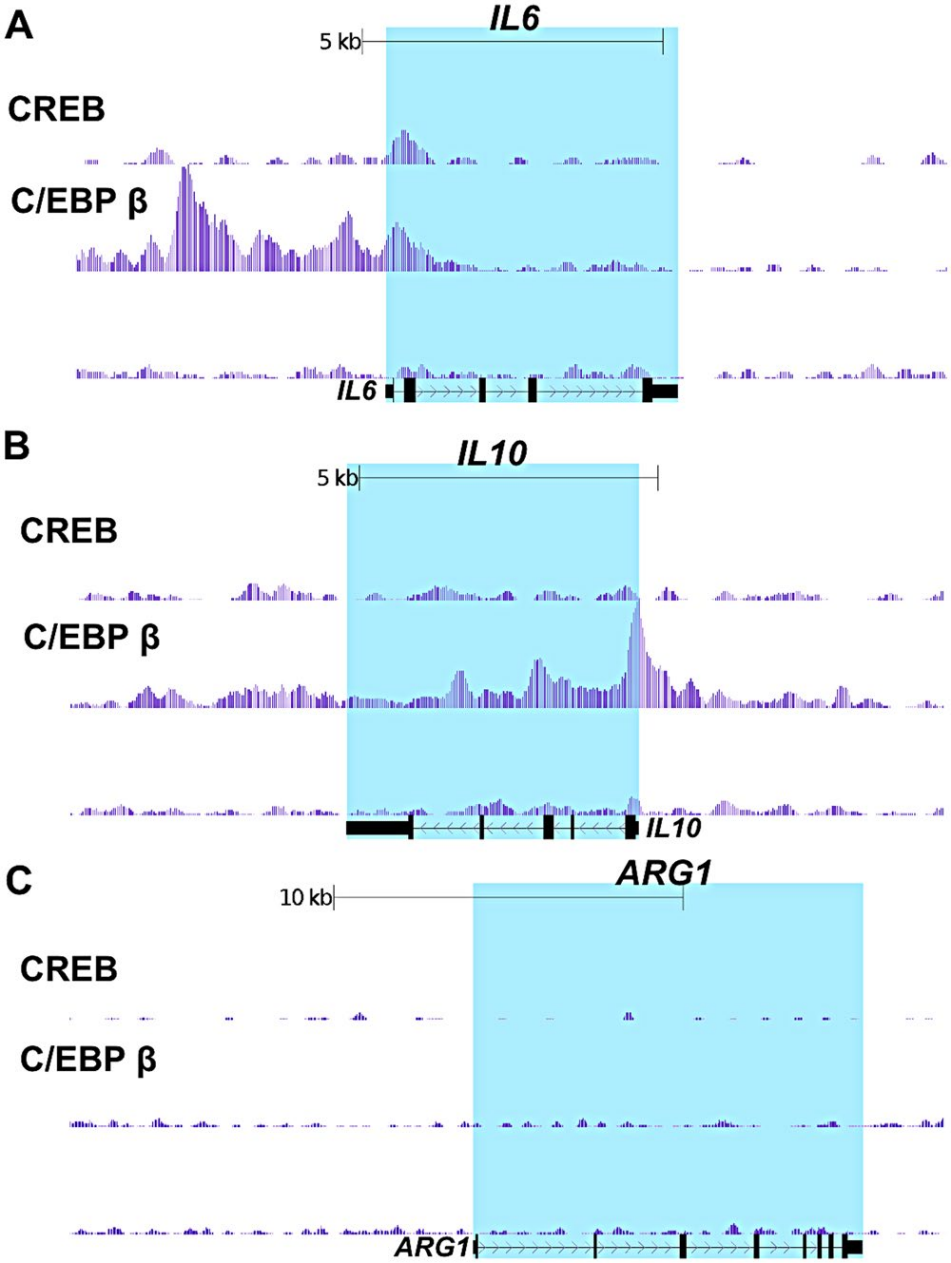
Mapping C/EBP β and CREB binding sites on the *IL6*, *IL10*, and *ARG1* genes in human PBMC. C/EBP β and CREB ChIP-seq analysis was performed on human PBMC pooled from 3 donors. Presented here are the UCSC genome browser views of C/EBP β and CREB binding the *IL6*, *IL10*, and *ARG1* genes.

Because C/EBP β responds to ET and is critical for mediating macrophage responses, in the next experiments a ChIP-seq approach was used to investigate C/EBP β localization on chromosomes in human PBMC. Using ChIP- validated antibodies specific for C/EBP β, we identified ∼35,400 peaks (i.e. putative sites of interaction with the chromosome). Motif analysis demonstrated the most intense peaks center on C/EBP β targeting DNA binding motifs. C/EBP β binding was detected in both the promoter and enhancer of the *IL6* gene and in the promoter of the *IL10* gene (Figs. 4A,B). Interestingly, C/EBP β was not detected in association with *ARG1* (Fig. 4C), suggesting a possible alternative mechanism of ARG1 regulation by C/EBP β. ChIP-seq was also performed on PBMC with ChIP-validated antibodies against CREB and ∼21,464 peaks were detected. Analysis of the most intense peaks found these centered on the CREB DNA binding motif (TGACGTCA). Examining the *IL10* and *ARG1* genes did not reveal CREB binding in proximity to these genes (Figs. 4B–C). For the *IL6* gene, CREB binding was observed within the gene at a position also occupied by C/EBP β (Fig. 4A).

### cAMP enhances recruitment of C/EBP β and CREB to a subset of PBMC genes

**Figure 5:**
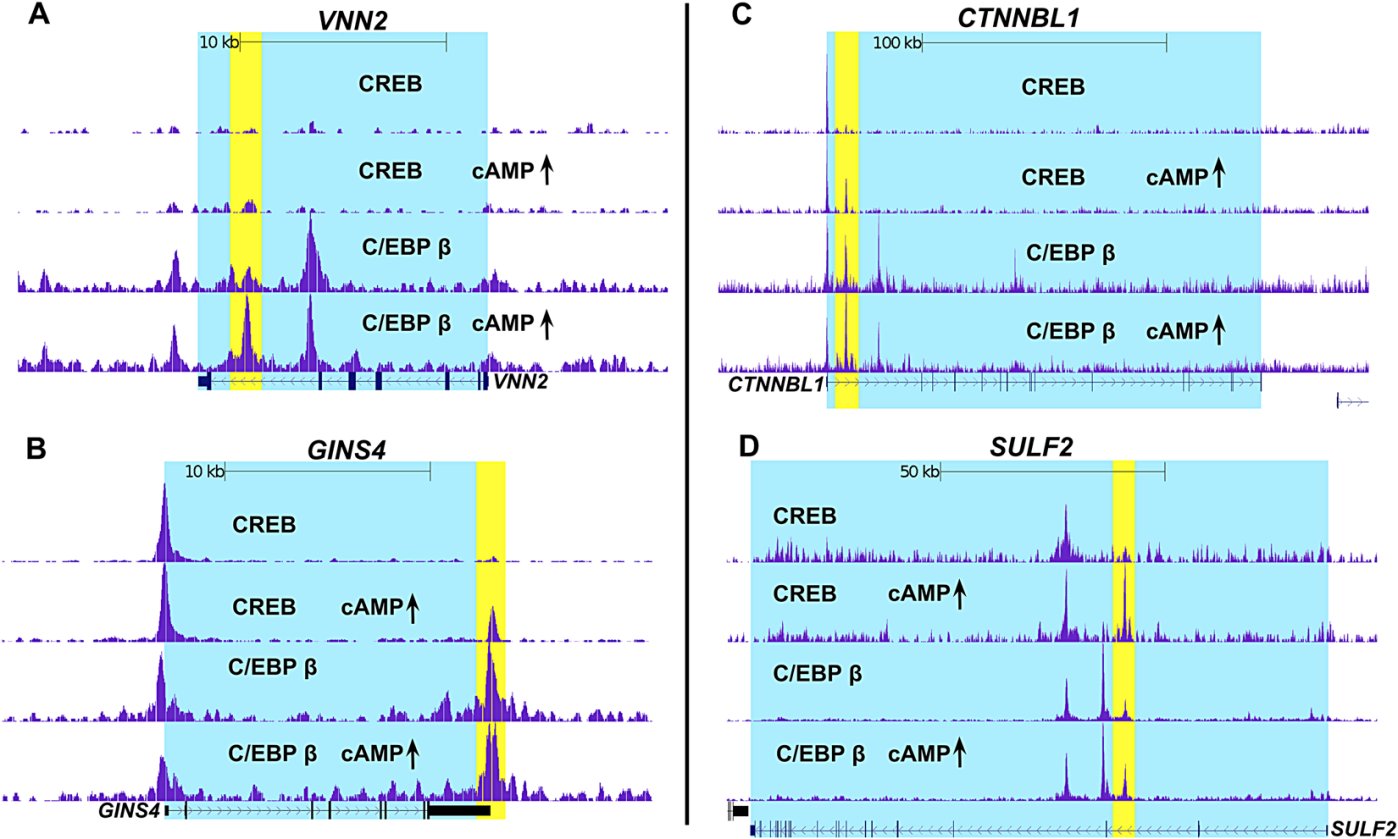
Enrichment of C/EBP β or CREB on genes during cAMP responses in human PBMC. Panel (A–D) C/EBP β and CREB ChIP-seq analysis were carried out on human PBMC pooled from 3 donors. For the data set examining C/EBP β, cAMP elevations were modeled by treating PBMC with 6MB-cAMP for 4 h. For the CREB data set, cAMP elevations were modeled by treating PBMC with ET (10 nM EF and 10 nM PA) for 4 h. Displayed here are the UCSC genome browser views of C/EBP β and CREB binding the genes.

**Figure 6:**
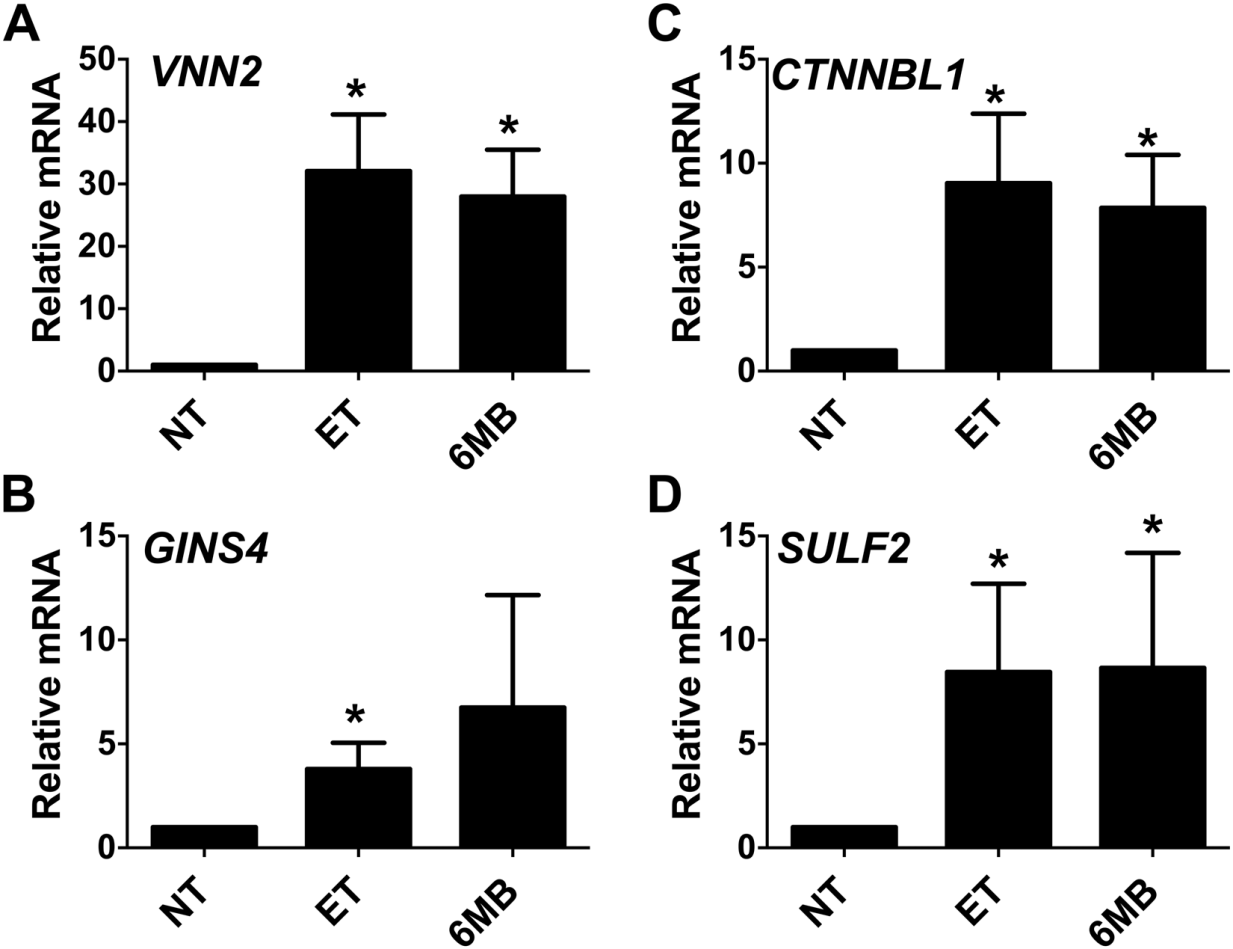
*VNN2, GINS4*, *CTNNBL1*, and *SULF2* are up regulated by cAMP in human monocytes. Panel (A–D) Human monocytes were isolated as described in the Methods. These cells were exposed for 6 h to 10 nM ET (10 nM EF and 10 nM PA) or 500 μM 6MB-cAMP. RT-qPCR was then used to quantify transcript levels of *VNN2, GINS4*, *CTNNBL1*, and *SULF2*. Bar graphs are representative of 3 independent experiments with monocytes derived from different donors. Error bars indicate mean ± S.D. Asterisks indicate significant different than control cells. *p < 0.05.

We also performed the ChIP-seq experiment on PBMC treated with cAMP elevating agents to determine if we could detect the recruitment of C/EBP β or CREB to DNA binding sites. As shown in Supplementary Table S1, few sites were found that recruited C/EBP β during elevated cAMP in PMBC, with only 141 regions enriched for C/EBP β under this treatment condition. In the case of CREB, cAMP elevations in PBMC also induced the recruitment of CREB to a limited number of genes with 249 regions enriched for CREB (Supplementary Table S2). Figure 5 shows some selected examples of genes that have C/EBP β and/or CREB recruited to putative enhancer regions after cAMP elevations in PBMC. As shown in Fig. 5A, cAMP induces C/EBP β, but not CREB, to bind the *VNN2* gene downstream (+17,355) of the transcription start site. Experiments next determined if the cAMP-dependent enrichment of C/EBP β at the *VNN2* gene in PBMC correlated with increased gene expression in purified human monocytes with increased cAMP. Thus, in human monocytes treated with ET or 6MB-cAMP, RT-qPCR results revealed robust expression of *VNN2* by cAMP (Fig. 6A). A common profile in these PBMC was the cAMP-dependent recruitment of CREB to regions that are constitutively occupied by C/EBP β. In Fig. 5B–D, examples of this phenomenon in PBMC are provide by 3 genes (*GINS4*, *CTNNBL1*, and *SULF2*). In these examples, cAMP increases result in CREB enrichment at sites downstream of the transcription start site (*GINS4* +*15,935; CTNNBL1* +7994; and *SULF2* +45,412) (Fig. 5B–D). As a demonstration of the possible consequence of recruiting CREB, transcript levels were evaluated in human monocytes and found that cAMP elevations (ET or 6MB-cAMP) induce the expression of *GINS4*, *CTNNBL1*, and *SULF2* (Fig. 6B–D).

### CREB and C/EBP β bind TLE promoters in human PBMC

**Figure 7:**
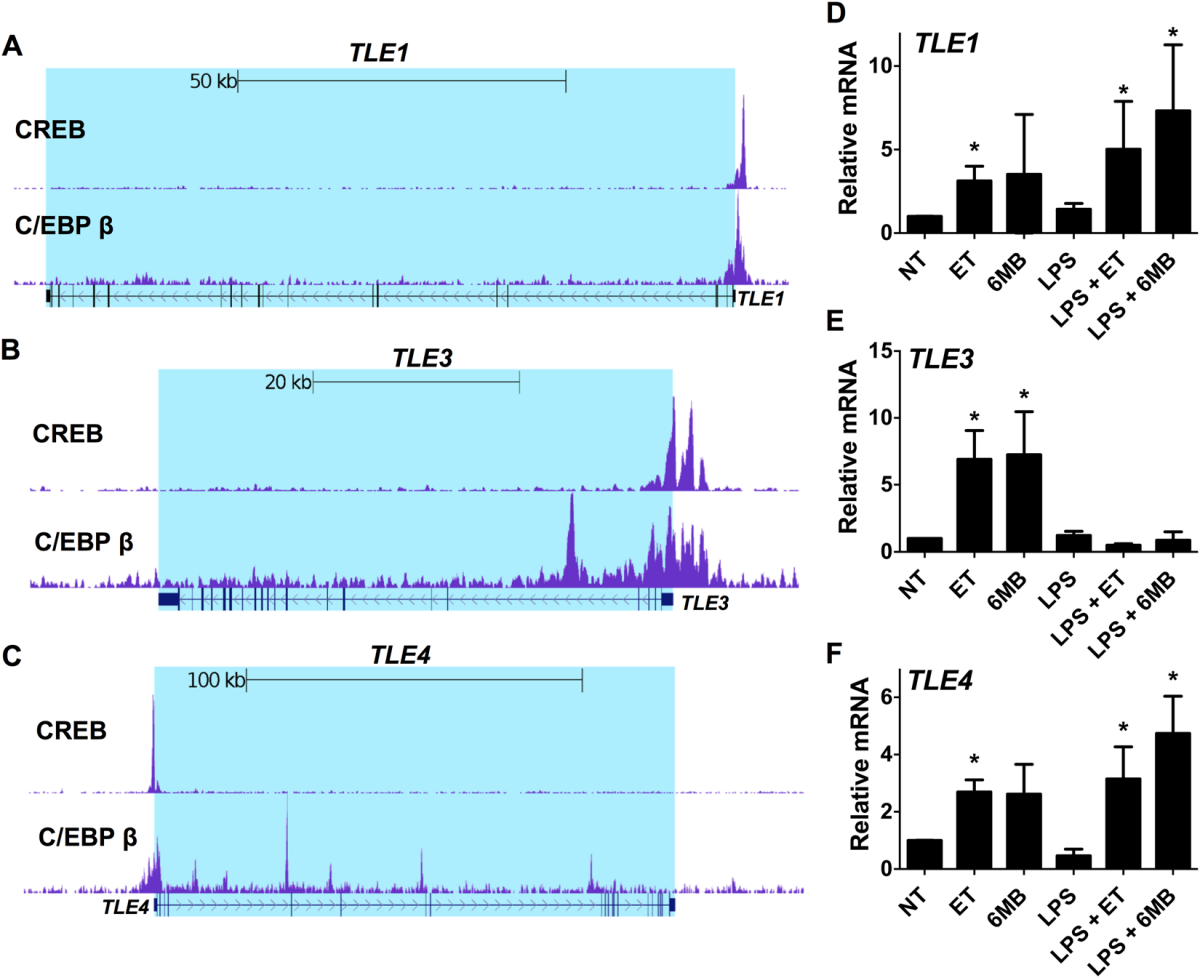
C/EBP β and CREB bind the promoter of TLE genes, and TLE genes are induced by cAMP in human monocytes. Panel (A–C) C/EBP β and CREB ChIP-seq analysis were performed on human PBMC pooled from 3 donors. Displayed here are the UCSC genome browser views of C/EBP β and CREB binding the *TLE1*, *TLE3*, and *TLE4* genes. Panel (D–F) Human monocytes were isolated as described in the Methods. These cells were exposed for 6 h to one of the following or a combination of the following: 10 nM ET (10 nM EF and 10 nM PA), 1 mM 6MB-cAMP, or 100 ng/ml LPS. RT-qPCR was then used to quantify transcript levels of *TLE1*, *TLE3*, and *TLE4*. Bar0 graphs are representative of 3 independent experiments with monocytes derived from different donors. Error bars indicate mean ± S.D. Asterisks indicate significant different than control cells. *p < 0.05.

The ChIP-seq analysis also identified genes with CREB and C/EBP β constitutively occupying promoter regions. *TLE1* had such a profile as shown by C/EBP β binding at position −295 and CREB binding at position −300 (Fig. 7A) in the absence of elevated cAMP. Interestingly, we have previously demonstrated that *TLE1* is induced by cAMP in human monocytes^5^ suggesting some cAMP responsive promoters are primed with cAMP transcription factors under basal conditions. This observation is also noteworthy because recent work by other groups has revealed that TLE1 is a negative regulator of inflammation^17,18^.

In addition to TLE1, the TLE family of transcriptional corepressors is composed of other members such as TLE3 and TLE4. As such, experiments next evaluated *TLE3* and *TLE4* to determine if C/EBP β or CREB bind the promoter of these genes in a similar fashion as *TLE1*. As shown in Fig. 7B–C, *TLE3* and *TLE4* had C/EBP β and CREB in their promoters. Transcript levels of *TLE3* and *TLE4* were also examined in human monocytes in response to elevated cAMP. As shown by RT-qPCR, both ET and 6MB-cAMP induced *TLE3* and *TLE4* transcripts similarly to *TLE1* (Fig. 7D–F). Considering the role of TLE1 in immunosuppression, we also asked whether activating inflammatory processes in monocytes could alter how cAMP impacts TLE expression. Thus, human monocytes were exposed to LPS in the presence and absence of ET or 6MB-cAMP and expression levels of *TLE1*, *TLE3*, and *TLE4* were measured. As shown in Fig. 7D,F, LPS treatment increased *TLE1* transcript levels and caused a modest increase in *TLE4* expression. Conversely, LPS completely reversed the cAMP-mediated induction of *TLE3* in human monocytes (Fig. 7E). Thus, while each of the three *TLE* genes contain C/EBP β and CREB in their promoter regions and can be modulate by cAMP, *TLE3* appears to differ in its susceptibility to modulation by LPS.

### PKA intersects with the non-canonical cAMP pathway through interactions with APC

**Figure 8:**
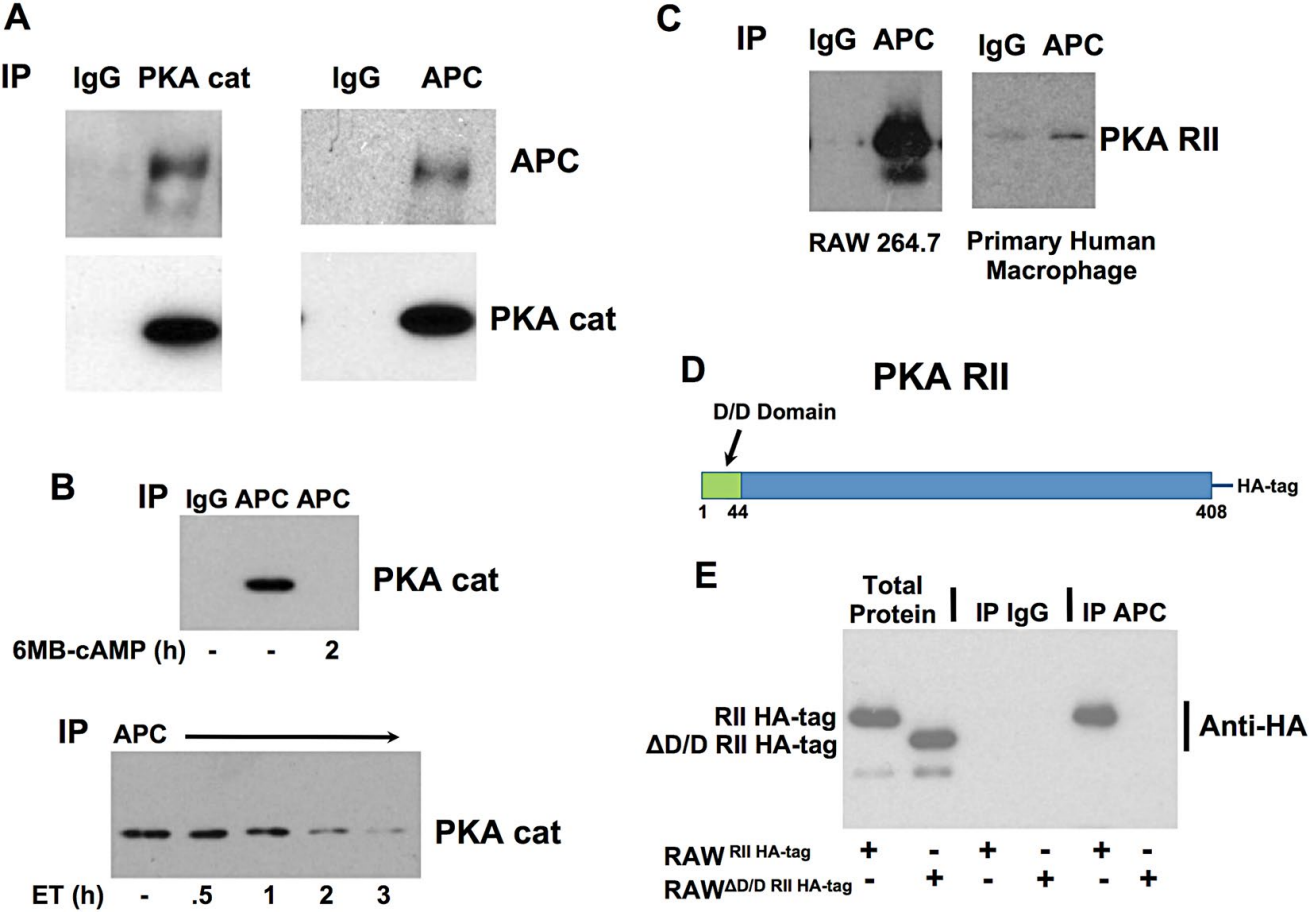
APC binds PKA and responses to cAMP. Panel (A) APC binds PKA cat in RAW 264.7 cells. Immunoprecipitations were executed with control rabbit IgG, rabbit IgG against APC, or rabbit IgG against PKA cat α. The subsequent immunoblots were probed with antibodies against APC or PKA cat α. Panel (B) Elevated cAMP in macrophages leads to the release of PKA cat α from APC. Immunoprecipitations were performed using protein extracts taken from RAW 264.7 cells exposed 1 mM 6-MB-cAMP or 10 nM ET. Immunoprecipitations were carried out with control rabbit IgG or rabbit IgG against APC. The immunoblots were probed with antibodies recognizing PKA cat α. Panel (C) APC binds PKA RII in RAW 264.7 cells and human monocytes. Immunoprecipitations were performed using protein extracts from RAW 264.7 cells or primary human macrophages using control IgG or IgG against APC, and immunoblots were probed with antibodies recognizing PKA RII. Panel (D) Domain layout of PKA RII highlighting the docking and dimerization (D/D) domain that is deleted from PKA RII. Panel (E) RAW 264.7 cells were transduced with a replication incompetent retrovirus containing RII HA-tagged or ΔD/D RII HA-tagged. Immunoprecipitations were then performed with control rabbit IgG or rabbit IgG against APC. The immunoblots were probed with antibodies recognizing the HA-tag.

In previous work, we found that C/EBP β was part of a cAMP responsive protein complex including APC and GSK-3^6^. Within this complex, GSK-3 phosphorylates and activates C/EBP β in an APC dependent manner^6^. Considering this, we were curious to know if components of the cAMP pathway such as PKA also associated with APC. Therefore, the catalytic subunit of PKA (PKA cat α) was immunoprecipitated from RAW 264.7 cells and the sample was probed using antibody to APC. In the reciprocal analysis, APC was immunoprecipitated and the sample was probed using antibody to the catalytic subunit of PKA. As shown in Fig. 8A, the results of this analysis suggest PKA and APC interact within the cell. Next an analysis was performed to determine if increased levels of cAMP could alter binding interactions between APC and PKA cat α. Thus, RAW 264.7 cells were treated with ET or 6MB-cAMP and binding interactions between APC and PKA cat α was examined. Interestingly when macrophages were treated with ET or 6-MB-cAMP, PKA cat α was released from APC as shown in the co-immunoprecipitation experiment in Fig. 8B.

To further define the binding mechanism between PKA and APC, interactions between APC and the PKA regulatory subunit (PKA R) were analyzed. In this experiment, an immunoprecipitation was performed that determined whether PKA RI or RII was able to bind APC. Results from this experiment demonstrated that the RII subunit of PKA bound APC (Fig. 8C) while the RI subunit did not bind APC. This interaction was observed in both RAW 264.7 cells (mouse macrophage cell line) and primary human macrophages (Fig. 8C). Because RII commonly binds to A-Kinase Anchor Proteins (AKAP), we tested the possibility that APC bound the RII through an AKAP like mechanism. As shown in Fig. 8D, PKA RII has a N-terminal docking and dimerization (D/D) domain responsible for binding AKAP. Thus, we determined if APC was able to bind a mutant form of the PKA RII that lacks the D/D domain (PKA RII ΔD/D). In these experiments, PKA RII and PKA RII ΔD/D were expressed in RAW264.7 cells by transducing these cells with a retrovirus containing a HA-tagged form of PKA RII or PKA RII ΔD/D. As shown in Fig. 8E, each form of HA-tagged RII expressed at comparable levels in transduced RAW 264.7 cells. Next, we immunoprecipitated APC from the cells transduced with HA-tagged RII or HA-tagged RII ΔD/D and then performed an immunoblot probing with antibodies against the HA-tag. As shown in Fig. 8E, HA-tagged RII was detected but HA-tagged RII ΔD/D was not in these APC immunoprecipitations. These data indicate that the D/D domain is necessary for PKA R II to bind APC, suggesting APC/PKA interactions are similar to AKAP/PKA interactions. Overall, these results indicate PKA, like other ET-modulated factors, interacts with APC and this may be a crucial site for localized targets of cAMP signaling.

## Discussion

While much is known about how *B. anthracis* as well as other pathogens elevate cAMP within host cells^2^, less is known about how cAMP reprograms cell such as macrophages. Many of the signaling activities of cAMP are carried out by the activation of PKA and the basic region leucine zipper transcription factors CREB and C/EBP β^4,6,10^. These cAMP effectors are used ubiquitously by cells, but limited information is available describing how these proteins affect macrophage phenotypes after cAMP is elevated during infection. Here, a multi-pronged approach was undertaken to examine 3 critical nodes of the vast cAMP signaling network in monocytic cells. First, CRISPR/Cas9 gene editing in a macrophage cell lines was used to examine the contributions of CREB and isoforms of C/EBP β to inflammatory responses and cAMP elevations (Figs 1–3). In the second group of studies, CREB and C/EBP β were analyzed by ChIP-seq in PBMC in order to capture the activity of these transcription factors in primary human cells (Figs 4–7). In the final group of experiments, data are provided describing an APC containing complex that interacts with PKA possibly providing a link between PKA and C/EBP β (Fig. 8). Collectively, these results indicate CREB and C/EBP β are modulated by inflammatory stimuli and cAMP in a complex system where the two factors share identical gene targets, but also work independently on other genes.

From our work examining macrophages deficient in CREB or C/EBP β, we found that C/EBP β appeared to contribute more prominently to dampening production of inflammatory cytokines and promoting expression of IL-10 and Arg1 (Figs 1–3). For example, macrophages lacking C/EBP β hyperexpress *Il6* while exhibiting a 50-fold reduction in *Il10* transcript levels in response to LPS (Figs 1, 2). This increase in IL-6 by C/EBP β loss was at multiple time points as shown by ELISA in Supplementary Fig. S1. Although Arg1 and IL-10 were not examined over a time course, we did examine these factors under a diverse set of conditions (LPS, ET, and IL-4/ET) and found a consistent, strong repression of these factors (Figs 1–3). This work also demonstrated that CREB exhibits a more modest influence over expression of markers of inflammation, which could be due to other CREB family members (ATF1, CREM) having activities that overlap with CREB^19,20^. Based on these findings, ET appears to exploit C/EBP β by activating the anti-inflammatory activities supported by this transcription factor.

Using CRISPR/Cas9 gene editing, we explored how the isoforms of C/EBP β contribute to inflammatory responses and cAMP elevations. The results indicate the 38 kDA form of C/EBP β is necessary for the induction of IL-10 and Arg1 while all 3 isoforms contribute to keeping LPS induced IL-6 in check (Figs 1–3). The 38 kDa isoform of C/EBP β is required for inducing IL-10 and Arg1 possibly because the 38 kDa isoform effectively interacts with chromatin modifiers such as the SWI/SNF complex^21^ and p300^22^ while the shorter isoforms (36 kDa and 20 kDa) lack the regions necessary for proper interactions with these factors. Findings from the ChIP-seq analysis also support the idea that C/EBP β is critical for the regulation of IL-10 and Il-6 (Fig. 4). This analysis detected C/EBP β at the promoter regions of the *IL6* and *Il10* genes suggesting these cytokine genes are primed to be activated or repressed through C/EBP β.

Results from the ChIP-seq also indicate several unexpected layers of complexity in the CREB, C/EBP β regulatory network. In contrast to past models describing CREB function and in agreement with recent ChIP-seq data^23,24^, few DNA regions exhibit cAMP dependent recruitment of CREB or C/EBP β. Most of the DNA binding regions is constitutively occupied by CREB, C/EBP β, or both. CREB appears to be more responsive than C/EBP β in regards to cAMP-inducible binding, as CREB was recruited to 249 regions and C/EBP β recruited to 149 regions (Supplementary Information). The cAMP-inducible binding of CREB and C/EBP β is located outside regions that are considered promoters and are most likely enhancers, which is observed with genes such as *CTNNBL1*, *SULF2*, *GINS4*, and *VNN2* (Fig. 5). Interestingly, two of these genes (*SULF2* and *VNN2*) lack CREB or C/EBP β binding prototypical promoters (Fig. 5). Because the ChIP-seq data is from PBMC, we determined if these 4 genes were also induced in purified human monocytes; therefore, we measured transcript levels and found that these 4 genes were induced in a cAMP dependent manner (Fig. 6). Of these 4 genes, *VNN2* is recognized as a factor involved in inflammation and leukocyte migration^25^ as well as can be upregulated by cAMP increases^26^.

The ChIP-Seq data in PBMC also corroborated our previous biochemical findings that increased cAMP leads to the up regulation of TLE1 in human monocytes. Both CREB and C/EBP β were found to bind at promoter regions of *TLE1* as well as the promoters of other TLE isoforms, TLE3 and TLE4 (Fig. 7). The binding of CREB and C/EBP β to these promoters was not induced by increased cAMP; however, we confirmed that TLE3 and TLE4 respond to cAMP in monocytes by demonstrating that transcript levels of these TLE isoforms were upregulated in purified human monocytes (Fig. 7). Transcript levels were also measured under inflammatory conditions created by LPS stimulation, and results revealed that cAMP-induced TLE1 and TLE4 were not reduced by LPS (Fig. 7). However, cAMP-induced TLE3 was reduced to basal levels after LPS stimulation, suggesting an unexplored layer of regulation in which LPS-induced signaling represses transcription of *TLE3* (Fig. 7). TLE are a family transcription corepressor proteins (TLE1-4) recruited to genes through interaction with DNA binding proteins such as Hes and TCF/LEF^27^. Recently other groups have discovered that TLE1 and TLE4 help control inflammation and are involved in anti-inflammatory phenotypes^17,18,28,29^. Our observations that TLE is upregulated by increased cAMP suggest that TLE contributes to the mechanism of cAMP mediated immunosuppression^5^.

In the final group of experiments, we examined the mechanism of how cAMP may regulate the activity of C/EBP β. Our previous work had shown that C/EBP β is part of a protein complex that included APC and GSK-3^6^; however, we were unsure how this complex connected to cAMP signaling. We were surprised to discover that APC was able to bind both the catalytic and regulatory subunits of PKA (Fig. 8). Further work demonstrated that APC demonstrated characteristics of a AKAP, such as releasing the catalytic subunit of PKA after cAMP stimulation and binding the regulatory subunit through the D/D domain (Fig. 8). APC is known to scaffold interactions between a range of signaling proteins and was recently linked to inflammatory activities^30^. Thus, APC functioning as a center for cAMP signaling and regulating inflammation is probable and a subject of future studies.

## Methods

### Institutional compliance

Experimental protocols, environmental and biological safety, and research involving materials from human subjects were reviewed and approved by the institutional biosafety committee and institutional review board. All experiments were carried out in accordance with relevant guidelines and regulations.

### Reagents

The membrane-permeable cAMP analog, N^6^-monobutyryladenosine 3′,5′-cyclic monophosphate (6MB-cAMP), was obtained from Biolog (Bremen, Germany). LPS (catalog number tlrl-eblps) was acquired from Invivogen (San Diego, CA). IL-4 (catalog number 214-14) was purchased from Peprotech (Rocky Hill, NJ). PA (catalog number 171E) and EF (catalog number 178a) were purchased from List Biological Laboratories (Campbell, CA).

### Cell culture and isolation of primary human cells

RAW 264.7 were obtained from the ATCC and cultured in DMEM with 10% FBS. Peripheral blood mononuclear cells (PBMC) were isolated from buffy coats derived from deidentified human whole blood obtained from the Oklahoma Blood Institute under a protocol categorized as exempt by the Institutional Review Board at the University of Oklahoma Health Sciences Center. To isolate PBMC, buffy coats were diluted in complete RPMI 1640 medium (10% FBS plus penicillin/streptomycin), and then applied to density gradient centrifugation with Histopaque 1077 (Sigma-Aldrich). In some experiments, monocytes were purified from PBMC using a monocyte isolation kit acquired from Miltenyi Biotec (Auburn, CA). This kit utilizes a negative selection method in which all non-monocytes are labeled with magnetic beads and separated from monocytes. The isolated monocytes were diluted into complete RPMI 1640 medium at a concentration of 1.0 × 10^6^ cells/ml and then exposed to experimental conditions. In other experiments, macrophages were cultured from the human PBMC. To culture human macrophages, monocytes were first isolated by adhesion to tissue culture plastic and the resulting monocytes were added to 6-well plates in RPMI 1640 media containing 7.5% heat inactivated autologous human fibrin-depleted plasma, penicillin/streptomycin, and 500 ng/ml M-CSF (Peprotech). The culture was continued for a week as the monocytes differentiated into macrophages and then used in immunoprecipitation experiments.

### Generation of cell lines with CRISPR/Cas9 gene editing

Lentivirus particles containing Cas9, gRNA, and a puromycin resistant gene (*pac* gene) were prepared by the following method using these 3 plasmids: pCMV-VSV-G (Addgene plasmid 8454 gift from Bob Weinberg)^31^ psPAX2 (Addgene plasmid 12260 gift from Didier Trono), and lentiCRISPRv1 (Addgene plasmid 52961 gift from Feng Zhang)^32^. With the lentiCRISPRv1 plasmid, gRNA directed towards CREB or C/EBP β were cloned into the plasmid. The sequences of the gRNA were based on the sequences used in the mouse GeCKO (Genome-Scale CRISPR Knock-Out) lentiviral pooled library^32^ and are as follows: CREB, TGTACTGCCCACTGCTAGTT; total C/EBP β, GCGCAGGGCGAACGGGAAAC; Δ38 C/EBP β, CGCGTTCATGCACCGCCTGC; and Δ38/36 C/EBP β, CGGCTTGGCGCCGTAGTCGT. These plasmids were cotransfected into 293FT cells (ThermoFisher Scientific; catalog number: R70007) using the calcium phosphate transfection method. 293FT cells were cultured in DMEM media containing 10% FBS and supplemented with 0.1 mM MEM Non-Essential Amino Acids, 1 mM sodium pyruvate and 2 mM L-glutamine. Forty-eight hours after transfection, lentivirus particles released into the media were harvested and used to transduce RAW 264.7 cells. Lentivirus transduction was accomplished by adding lentivirus particles plus 8 μg/ml Polybrene to cells plated at 2.5 × 10^4^ cells/well in a 12-well plate and then centrifuging the lentivirus mixture with cells for 2 h at 1200 × *g* at 25 °C. After incubating the cells with lentivirus overnight at 37 °C, the lentivirus particles were removed and the cells were placed under antibiotic selection with 3.5 μg/ml of puromycin for 3–4 days to kill non-transduced cells. Single cell colonies were then isolated and grown in the presence of puromycin. The colonies were then screened for CREB or C/EBP β gene disruption by PCR of genomic DNA and immunoblotting.

### Reverse Transcriptase qPCR Analysis

RNA was isolated from RAW 264.7 cells or human monocytes and then converted to cDNA in reverse transcription reactions using SuperScript III (Invitrogen). Equal amounts of cDNA for each sample were combined with a SYBR Green PCR master mix (Qiagen) and gene-specific primers. Amplification reactions were then performed with an Applied Biosystems 7500 real-time PCR system. Relative changes in levels of the mRNA of the gene of interest were compared with the levels of mouse *Actb* or human *ACTB* mRNA using the 2^−ΔΔCt^ method.

### Immunoblot

Total proteins were extracted from RAW 264.7 cells by removing culture medium and then adding 4 °C lysis buffer containing 1% SDS, 50 mM Tris (pH 7.4), 5 mM EDTA, and a protease inhibitor mixture (Sigma, catalog no. P8340). The cells were incubated in this lysis buffer on ice for 15 min, passed through a 22-gauge needle 10 times, and centrifuged for 5 min at 20,000 × *g*. For immunoblot analysis, these proteins extracts (10 μg/well) were combined with sample buffer (62.5 mM Tris-HCl (pH 6.8), 2% SDS, 10% glycerol, 5% β-mercaptoethanol, and 0.001% bromphenol blue) and heated at 95 °C for 7 min. Proteins were then separated using 10–12% SDS-PAGE and transferred to a PVDF membrane by electroblotting. The membrane was blocked with 5% nonfat milk in a wash buffer consisting of 20 mM Tris-HCl (pH 7.5), 100 mM NaCl, and 0.1% Tween 20. The membranes were then probed with rabbit monoclonal antibody against CREB (Cell Signaling Tech; catalog number 9197), rabbit antibodies against C/EBP β (Santa Cruz Biotechnology; catalog number sc-150), rabbit monoclonal antibody against PKA cat (Cell Signaling Tech, catalog number 5842), rabbit antibodies against PKA RII (Santa Cruz Biotechnology; catalog number sc-908 and sc-909), mouse monoclonal antibody against APC (clone FE9; Millipore Sigma; catalog no. OP44), rabbit monoclonal antibody against HA-tag (Cell Signaling Tech, catalog number 3724) or a mouse monoclonal antibody against GAPDH (Abcam; catalog no. ab8245). The membrane was then washed and incubated with a secondary antibody conjugated to horseradish peroxidase. The immunoblots were developed with an enhanced chemiluminescent protein development system (GE Healthcare) and exposed to film or imaged with a ChemiDoc MP Imaging System (Bio-Rad).

### ChIP-Seq and data processing

PBMC were plated at 2 million cells per ml in RPMI containing 10% FBS, 100 units/ml penicillin, and 100 μg/ml streptomycin. These cells were then treated with 10 nM ET (10 nM EF and 10 nM PA) or 500 μM 6MB-cAMP for 4.5 h and then isolated by centrifugation followed by snap freezing in liquid N_2_ as recommended by Active Motif. For each condition, 60 million PBMC were prepared from 3 donors (20 million PBMC per donor). ChIP was performed by Active Motif with antibodies validated for ChIP against CREB (Cell Signaling Tech; catalog number 9197) or against C/EBP β (Santa Cruz Biotechnology, catalog number sc-150). After ChIP DNA libraries were constructed, 75-nt sequence reads were generated by Illumina sequencing.

### Coimmunoprecipitation

For the described coimmunoprecipitation analyses, cells were lysed by incubating for 5 min in lysis buffer (20 mM HEPES (pH 7.9), 350 mM NaCl, 30 mM MgCl_2_, 10% glycerol, 0.5% Nonidet P-40, 200 μM DTT, and protease inhibitor mixture), passing the lysates through a 22-gauge needle 10 times, and then centrifuging the lysates at 18,000 × *g* for 5 min. Next, 500 μg of total protein from the lysate was diluted into a binding buffer (20 mM HEPES (pH 7.9), 30 mM MgCl_2_, 10% glycerol, 0.2% Nonidet P-40, 200 μM DTT, and protease inhibitor mixture) to produce a final volume of 300 μl. The following antibodies were then added to the immunoprecipitations: rabbit antibodies against APC (Santa Cruz Biotechnology, catalog number sc-896), rabbit monoclonal antibody against PKA cat (Cell Signaling Tech, catalog number 5842), or control rabbit IgG (Santa Cruz Biotechnology, catalog number sc-2027). The immunoprecipitations were subsequently incubated for 2 h at 4 °C. Then, protein G-conjugated magnetic beads were added, and the incubation was continued for an additional 30 min. After removing the input protein, the magnetic beads were washed three times in 200 μl of binding buffer, and the immunoprecipitated proteins were eluted and subjected to immunoblot analysis.

### Expression of PKA RII in RAW 264.7 cells

PKA RII is the product of the *Prkar2a* gene. Thus, PCR was used to amplify the *Prkar2a* gene from mouse cDNA and to fuse DNA sequence to the gene in order to produce a HA tag on its C-terminal (RII HA-tagged). PCR primers were also designed to amply the portion of the *Prkar2a* gene that does not produce the N-terminal D/D domain (ΔD/D RII HA-tagged). Each of these PCR products was cloned into the pENTR/D-TOPO vector (Invitrogen) and subsequently transferred into expression vectors through a recombination reaction using LR Clonase (Invitrogen). The retrovirus expression vector used to express RII HA-tagged or ΔD/D RII HA-tagged was pQCXIB (Addgene plasmid 17487, originating from E. Campeau)^33^. For retrovirus-mediated expression of these proteins, pQCXIB along with murine leukemia virus gag/pol and vesicular stomatitis virus G expression plasmids were each cotransfected into 293 T cells (cultured in DMEM with 10% FBS) using the calcium phosphate transfection method. Forty-eight hours after transfection, retroviral particles were harvested and used to transduce RAW 264.7 cells. For RAW 264.7 cell transduction, the retrovirus particles plus 8 μg/ml Polybrene were added to cells plated at 5.0 × 10^4^ cells/well in a 12-well plate. The plate containing the cell and the retrovirus mixture were then centrifuged for 2 h at 1200 × *g* at 25 °C and placed in the tissue culture incubator overnight. Retrovirus was then removed and replaced with fresh medium containing 2 μg/ml of blasticidin in order to kill non-transduced cells.

## Electronic supplementary material

Supplementary Information

Supplementary Table S1

Supplementary Table S2
