# Supplementary material for: Unique, Intersecting, and Overlapping Roles of C/EBP β and CREB in Cells of the Innate Immune System
Source: Sci Rep. 2018 Nov 16;8:16931. doi: 10.1038/s41598-018-35184-y (PMC6240029; doi:10.1038/s41598-018-35184-y)
Supplement: Supplementary file 1 — Supplementary Information [file 41598_2018_35184_MOESM1_ESM.pdf]

Supplementary Information

Unique, Intersecting, and Overlapping Roles of C/EBP β and CREB in Cells of the Innate Immune System

Jason L. Larabee, Garrett Hauck, and Jimmy D. Ballard*

Department of Microbiology and Immunology, The University of Oklahoma Health Sciences Center, Oklahoma City, OK 73190

*Corresponding Author: Jimmy D. Ballard, The University of Oklahoma Health Sciences Center, Department of Microbiology and Immunology, BMSB: 1053, 940 Stanton L. Young Blvd. Oklahoma City, OK 73104. Phone: 405-271-2133. E-mail: jimmy-ballard@ouhsc.edu

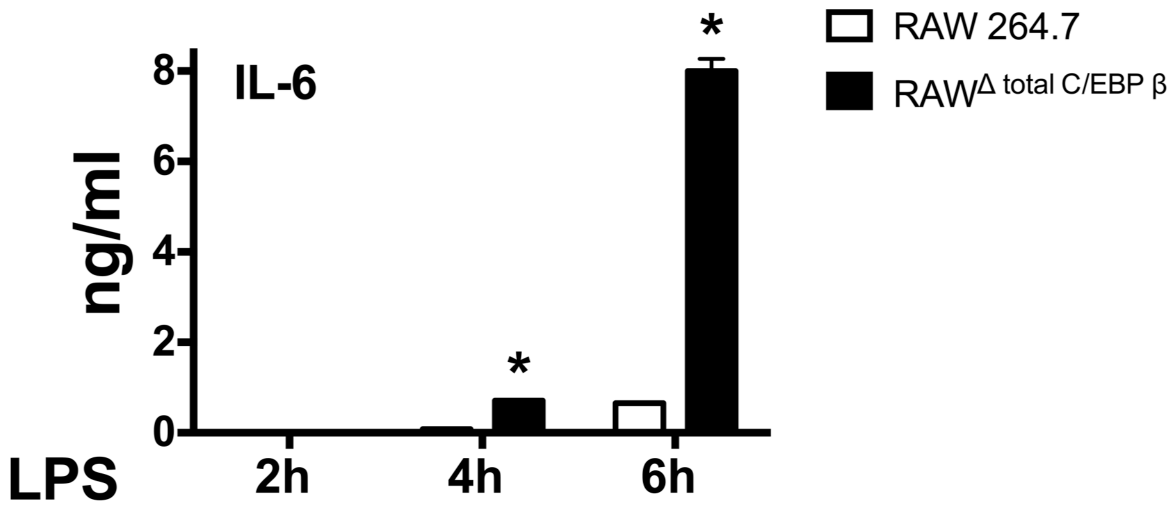

Supplementary Fig. S1. Absence of C/EBP β expression increases the secretion of IL-6 from macrophages. RAW 264.7 cells were transduced with lentivirus containing Cas9 and gRNA directed against C/EBP β. Following transduction, clones were selected that do not express any of the 3 forms of C/EBP β (RAW$^{\Delta \text{total C/EBP}\beta}$). RAW$^{\Delta \text{total C/EBP}\beta}$ cells and control RAW 264.7 cells were exposed to 1 μg/ml LPS for times indicated on the bar graph. IL-6 secreted into culture media was quantified by ELISA using a kit from Thermo Fisher Scientific. (Catalogue number: 88-7064-22). These data are presented as mean (n = 3) ± S.D. Asterisks indicate significant different than control cells. *, $p < 0.001$.
